# The completeness of intervention descriptions in published NIHR HTA funded trials: a cross sectional study

**DOI:** 10.1186/1745-6215-14-S1-O27

**Published:** 2013-11-29

**Authors:** Lisa Douet, Ruairidh Milne, Sydney Anstee, Fay Habens, Amanda Young, David Wright

**Affiliations:** 1 NETSCC, University of Southampton, Southampton, UK

## Objectives

The objective of this study is to assess whether NIHR HTA funded randomised controlled trials (RCTs) published in Health Technology Assessment journal were described in sufficient detail to replicate in practice.

## Methods

A checklist for assessing intervention descriptions was applied to NIHR HTA funded RCTs published in Health Technology Assessment. The checklist was piloted twice on a sample of 10 reports. Kappa scores were generated to assess agreement in the checklist application. The checklist was modified and applied to all 98 NIHR HTA funded single trial RCTs published in the journal from January 1999 - March 2011. Three assessors independently applied the checklist. Disagreements in scoring were discussed in the team; differences were then explored and resolved.

## Results

Components of the intervention description were missing in 68 / 98 (69.4%) reports. Baseline characteristics and descriptions of settings had the highest levels of completeness with over 90% of reports complete. Reports were less complete on patient information with 58.2% of the monographs having an adequate description. Intervention descriptions were more complete for drug interventions than non-drug interventions with 33.3% and 30.6% levels of completeness respectively. Only 27.3% of RCTs with psychological interventions were deemed to be complete, although numbers were too small for differences to be significant statistically.

## Conclusions

Ensuring the replicability of a study intervention is an essential part of adding value in research. Research funders need to ensure transparency in the reporting of interventions, methods and findings and their responses to identified areas of improvement.

